# Cardiorespiratory Response to Moderate Hypercapnia in Female College Students Expressing Behaviorally Inhibited Temperament

**DOI:** 10.3389/fnins.2020.588813

**Published:** 2020-11-13

**Authors:** Paul F. Martino, Daniel P. Miller, Justin R. Miller, Michael T. Allen, Denise R. Cook-Snyder, Justin D. Handy, Richard J. Servatius

**Affiliations:** ^1^Biology Department, Carthage College, Kenosha, WI, United States; ^2^Department of Physiology, Medical College of Wisconsin, Milwaukee, WI, United States; ^3^Neuroscience Department, Carthage College, Kenosha, WI, United States; ^4^School of Psychological Sciences, College of Education and Behavioral Sciences, University of Northern Colorado, Greeley, CO, United States; ^5^Naval Submarine Medical Research Laboratory, Groton, CT, United States; ^6^United States Department of Veterans Affairs, Syracuse VA Medical Center, Syracuse, NY, United States; ^7^Department of Psychiatry, State University of New York Upstate Medical University, Syracuse, NY, United States

**Keywords:** diatheses, temperament, stress, anxiety, heart rate variability, SDNN, vagal activity

## Abstract

Behaviorally inhibited (BI) temperament is marked by heightened behavioral sensitivity to environmental threats. The degree to which threat sensitivity is reflected in cardiorespiratory responses has been relatively unexplored. Female college students were exposed to modest hypercapnia (7.0% CO_2_) or ambient air (AA) while engaging in a computerized task with cued reinforcement features. All physiological variables except for blood pressure were processed in 4 min epochs corresponding to pre-exposure, exposure, and post-exposure. Primary respiratory measures were respiratory frequency (f_b_), tidal volume (V_T_), and minute ventilation (V_E_). Electrocardiograms (ECGs) were processed using ARTiiFACT software with resultant heart rate variability (HRV) measures in the frequency domain and time domain. Consistent with the literature, modest hypercapnia increased V_T_, F_b_, and V_E_. No differences in respiratory parameters were detected between BI and non-behaviorally inhibited individuals (NI). For HRV in the time domain, RMSSD and NN50 values increased during CO_2_ inhalation which then returned to pre-exposure levels after CO_2_ cessation. Hypercapnia increased high frequency (HF) power which then recovered. BI exhibited reduced low frequency (LF) power during the pre-exposure period. For NI, LF power reduced over the subsequent phases ameliorating differences between BI and NI. Hypercapnia improved the task performance of BI. This is the largest study of female reactivity to hypercapnia and associated HRV to date. In general, hypercapnia increased time domain HRV and HF power, suggesting a strong vagal influence. Those expressing BI exhibited similar respiratory and HRV reactivity to NI despite inherently reduced LF power. Although 7% CO_2_ represents a mild challenge to the respiratory and cardiovascular systems, it is nonetheless sufficient to explore inherent difference in stress reactivity in those vulnerable to develop anxiety disorders.

## Introduction

Anxiety disorders and posttraumatic stress disorder (PTSD) are best understood as a dynamic interaction of aversive experiences in those with inherent vulnerabilities to develop anxious states (Barlow, 2000; Chasiropoulou et al., 2019). A learning diathesis model accentuates individual differences in associative learning as a final common path to pervasive avoidance (Servatius et al., 2008; Beck et al., 2010; Allen et al., 2019), a core feature of anxiety disorders (Mellick et al., 2019), and PTSD (O’Donnell et al., 2007). The process by which avoidance develops may be through the individual differences in perceived aversiveness, the associativity of aversive experiences/ideations or both.

### Behaviorally Inhibited Temperament as a Vulnerability

Behaviorally inhibited (BI) temperament is one such vulnerability. BI is defined as a personality disposition marked by extreme withdrawal in the face of novel non-social or social challenges (Gladstone and Parker, 2005), with numerous studies linking BI to anxiety disorders (Biederman et al., 2001; Gladstone et al., 2005) and PTSD (Myers et al., 2012a,b; Servatius et al., 2017; Handy et al., 2018). Those individuals with BI temperament display associative learning biases, evident as facilitated acquisition of the classically conditioned eyeblink responses in adolescents (Holloway et al., 2012; Caulfield et al., 2015), adults (Allen et al., 2014, 2016), active duty military (Handy et al., 2018), and veterans (Myers et al., 2012a). The psychophysiological basis of associative learning biases in BI individuals is unclear. One line of reasoning focuses on attentional processes as a predisposing factor in learning (Mcauley et al., 2009). Those with BI temperament have difficulty disengaging attention from novel stimuli or stimuli associated with threat or distress (Pérez-Edgar et al., 2010; Ricart et al., 2011; Fox and Pine, 2012; Holloway et al., 2012; White et al., 2017).

### HRV as a Physiological Source of Vulnerability

A common substrate for hypervigilance and associative learning biases is the autonomic nervous system (Friedman, 2007), composed of the sympathetic and parasympathetic branches. Heart rate variability (HRV) is a means of assessing contributions of these two branches through decomposition of successive beat-to-beat variations in heart rate (peak R wave to R wave within the QRS complexes of electrocardiograms) (Berntson et al., 1997). HRV is partitioned in time and frequency domains. For the frequency domain, accepted bands are low frequency (LF; 0.04–0.15 Hz) and high frequency (HF; 0.15–0.4 Hz), the latter corresponding to the vagal influences on respiration (i.e., respiratory sinus arrhythmia). In the time domain, three measures are commonly evaluated: the standard deviation of normal to normal (NN) intervals (SDNN), root mean square of successive differences (RMSSD) between normal heartbeats, and the number of pairs of successive NNs that differ by more than 50 ms (NN50). Decreased HRV is evident as reduced LF power and increased HF power in the frequency domain and greater SDNN, RMSSD, and NN50 in the time domain. In general, better sustained attention or vigilance (Luque-Casado et al., 2016) and facilitated associative learning (Tapp et al., 1997) is associated with decreased HRV.

Further, temperaments with inhibitory features have inherently low HRV. For example, individuals expressing harm avoidance display reduced low frequency power (Puttonen et al., 2008; Huang et al., 2013). Moreover, distressed (Type D) personality, a combination of negative affect and social inhibition linked to cardiovascular disease, has been reported to have low HRV (Martin et al., 2010; Bibbey et al., 2015). Both harm avoidance (Allen et al., 2017) and Type D personality (Servatius et al., 2017; Allen et al., 2018; Handy et al., 2018) are highly correlated with BI temperament. Thus, it is reasonable to expect BI to have inherently reduced HRV or alternatively greater reductions of HRV from exposure to challenges.

### Experimental Induced Hypercapnia as a Challenge to HRV

Experimentally induced hypercapnia has a long history as a potent psychophysiological challenge. Panic-like responses are elicited by high concentrations of CO_2_ delivered in bolus (e.g., 35% CO_2_) (Van Den Hout et al., 1987; Perna et al., 2003) or lower concentrations (e.g., 5% CO_2_) delivered over extended exposure (e.g., 20 min) (Gorman et al., 1988; Bystritsky et al., 2000; Valenca et al., 2002). Such challenge parameters are quite demanding and may narrow the range in which to observe differences in those vulnerable to anxiety disorders.

Lower levels of CO_2_ delivered over shorter durations are more tolerable and produce gradations in demands placed on the system to adjust to hypercapnia from simple increases in tidal volume (V_t_) (Brown et al., 2014) to increased V_t_ accompanied by elevated HR (Tzeng et al., 2007). Even at mild levels of hypercapnia, the attendant acidosis drives deeper breathing through central chemoreceptors (Brown and Howden, 2008a). In terms of HRV, mild hypercapnia increases HF power (Brown et al., 2007, 2014; Tzeng et al., 2007). Although HF HRV is closely linked to vagal activity and respiratory sinus arrhythmia (RSA) in general, increased HF power secondary to hypercapnia may be dissociated from RSA (Brown et al., 2014). To date, time domain measures of HRV have been unaffected by mild hypercapnia (Brown et al., 2007). Thus, mild hypercapnia is potent enough to affect both respiratory parameters and HRV, particularly in the frequency domain.

### The Present Study

The present study examined respiratory and cardiovascular reactivity to mild hypercapnia as a function of BI temperament. Hypercapnia was expected to increase HF power. The Adult Measure of Behavioral Inhibition (AMBI) (Gladstone and Parker, 2005) was used to classify individuals as either BI or non-inhibited (NI). Those expressing BI were expected to exhibit reduced HRV. We further expected BI individuals to express more reactivity to mild hypercapnia. After a baseline period, participants engaged in a computerized task that requires attention; those expressing BI tend to perform better than NI on the task. Onset and terminations of the computer task were contemporaneous with introduction of hypercapnia and its return to ambient air (AA). The computerized task was used as a behavioral indicant of stress reactivity inasmuch as attention to hypercapnic challenge could be manifest as poorer performance on the task through divided attention. While male BI participants tend to perform better than male NI participants, BI females tend to be similar to NI females (Sheynin et al., 2014). Further, females are largely underrepresented in the study of mild hypercapnia and HRV (Brown et al., 2007; Tzeng et al., 2007). Therefore, we restricted this initial study to female participants.

## Materials and Methods

### Participants and Recruitment

Undergraduate volunteers were recruited at Carthage College, Kenosha, WI, United States. Ninety-nine females with an age range of 18–22 years voluntarily completed the study. Participants with a self-reported history of psychiatric illnesses, neurological disorders, cardiac disorders, or respiratory illnesses were excluded from the study. Participants were asked to refrain from drinking alcohol or chewing, smoking, or vaping any tobacco products for 24 h prior to the start of the study. Additionally, participants were asked to refrain from eating, exercising, or drinking caffeinated beverages for 2 h prior to the study. All eligible participants completed an informed consent agreement upon arrival for their scheduled study appointments and were given the opportunity to ask questions before initiating study procedures. Participants were not compensated for their participation, but received partial credit toward academic research requirements. The study was approved by the Institutional Review Board of Carthage College (IRB Approval #: 1133068-4) in compliance with all applicable Federal regulations governing protection of human subjects.

### Experimental Design

Participants were randomly assigned to receive either room ambient air (AA groups) through the breathing apparatus or 7% CO_2_ (7% CO_2_ groups). There were three phases: Baseline, Exposure, and Recovery. The Exposure and Recovery phases were 4 min each. The Baseline period was 15 min, but only the last 4 min were evaluated to equate with the other two phases for all physiological parameters. The exposure groups were characterized as NI or BI, as elaborated below. Thus, a 2 × 2 design was constructed: AA-NI (*N* = 29), AA-BI (*N* = 16), 7% CO_2_- NI (*N* = 32), and 7% CO_2_-BI (*N* = 21). During informed consent, participants were advised that exposure to hypercapnia was a possibility, but subjects were otherwise blind to assignment. With respect to behavioral inhibition, investigators were blind to the condition.

### Scales

All participants completed the Adult Measure of Behavioral Inhibition (AMBI) which consists of 16 items probing aspects of BI temperament (Gladstone and Parker, 2005; Gladstone et al., 2005). Items assess the degree to which participants exhibit inhibited or avoidant behaviors in new or unfamiliar social and non-social situations on a 3-point Likert-type scale. Possible scores range from 0 to 32 with higher scores indicating higher levels of BI. Consistent with previously established methodology (Allen et al., 2014; Servatius et al., 2017; Handy et al., 2018), individuals were classified as NI or BI based on a cut score of 15.5.

### Computerized Task

Participants were presented with a computerized game to measure attention and performance during modest hypercapnia. The task was adapted from a spaceship game in which a participant defends a base from incoming spaceships which drop bombs, which has been more completely described elsewhere (Sheynin et al., 2015). The software was programmed in SuperCard version 3.7.1 (Solutions Etcetera, Pollock Pines, CA, United States) and presented on a Macintosh iMac computer. The keyboard was masked except for three keys labeled FIRE, LEFT, and RIGHT, which the subject could use to enter responses. Briefly, a participant began with 325 points which could increase or decrease based on performance. The game began with exposure and lasted 8 min.

### Gas Preparation, Delivery, Measurement

The custom system for gas preparation and delivery employed in the present study was previously described (Miller et al., 2018). Ambient air (AA) was mixed with medical grade CO_2_ using a 13.5 L respirometer/spirometer (P-1300, Warren E. Collins, Inc., Braintree, MA, United States) to achieve a concentration of 7% CO_2__._ This was verified through capnography using an O_2_CAP O2 and CO_2_ analyzer (Oxigraf, 07-7021). Once the gases were mixed, the mixture was transferred from the respirometer/spirometer to 300 g weather balloons, which could store up to 1600 L of gases for at least 1 h without any change in the concentrations of gases.

### Experimental Procedures

Data collection occurred during one of two 1-h time slots at 1400 and 1500 h 5 days a week (Monday–Friday). All participants were seated in the upright position in front of a computer desk which displayed a computerized task described above. Upon signing informed consent, blood pressure measurements were taken while seated upright with both arms resting on the deck in front of them, using a Leader fully automated blood pressure monitor (BP-3AG1-1PLDR, Cardinal Health). Subjects were outfitted with a face mask system that covered both the mouth and the nose and was connected to a two-way non-rebreathing valve and pneumotach (Model 3813, Hans Rudolph, Shawnee, KS, United States). Participants were also instrumented for ECG collection. Baseline acclimation to the apparatus was 15 min. During the acclimation period participants completed the demographic data and scales. At the end of the baseline period, the computerized task began. The task period was 8-min. During the first 4-min task period participants were exposed to AA or 7% CO_2_. During the last 4-min task period all participants were exposed to AA. Upon the task completion a second blood pressure measure was obtained.

### Respiration and ECG Collection and Processing

Airflow as minute ventilation (V_E_; L/min), breathing frequency (fb; breaths/min), and tidal volume (V_T_; L) were all measured with an airflow transducer and digital data acquisition module (Bio Pac MP36 and MP150, Goleta, CA, United States).

Three-lead electrocardiogram (ECG) was collected at 100 Hz with BIOPAC models (ECG 100C and MEC 110C) and digital data acquisition module (MP150). ECGs were extracted for the 4 min prior to start of task, 4 min of exposure (7% CO_2_ or AA), and 4 min of recovery. Subjects without complete records for the all three 4-min epochs were excluded from analysis. ECG was processed with ARTiiFACT software (Kaufmann et al., 2011). Interbeat intervals were computed from identified and visualized R waves. Artifacts were identified through Berntson detection and cubic spline correction. HRV parameters were computed with standard values of 4 Hz interpolation rate and 50% window overlap with standard frequency bands 0.04, 0.15, and 0.4 parameters. The very low frequency band (0.04–0.15 Hz) was otherwise ignored. Consistent with the literature, the normalized value of power (nu) as well as the natural log (ln) of raw power of LF and HF were analyzed to interpret power changes attributable to a particular band (Quintana and Heathers, 2014; Quintana et al., 2016). nuLF and nuHF are complementary, thus analysis of one is sufficient to understand power has changed with experimental conditions, but still not *specific* to either band. Analysis of the raw power is necessary to understand the nature of changes in nuLF/nuHF (Heathers, 2014).

### Analytic Approach

All statistical analyses were performed in IBM SPSS Statistics for Windows, version 26 (IBM Corp., Armonk, NY, United States) and R (R Core Team, 2020, Vienna, Austria). Respiratory measures and HRV measures were subjected to separate 2 (Exposure; AA vs. CO_2_) × 2 (Temperament; NI vs. BI) × 3 [Phase; Baseline (B), Exposure (E), and Recovery (R)] mixed analyses of variance (ANOVAs) with *p* < 0.05. Blood pressure measured prior to baseline recording and after recovery was analyzed with Exposure × Temperament × Time (pre vs. post) mixed ANOVA. Behavioral performance was analyzed with Exposure × Temperament multivariate ANOVA.

## Results

### Respiratory Parameters

Breathing frequency increased in all participants during the Exposure phase, which corresponded with the start of the computerized task. Breathing frequency increased from 14 breathes/min during baseline to 16–17 breathes/min during the Exposure and Recovery phases (See Table 1). The main effect of Phase was significant, *F*(2,178) = 87.3; η_p_^2^ = 0.58, *p* < 0.001. As expected, hypercapnia specifically increased V_T_, V_E_, and V_I_. Exposure × Phase interactions were appear for both V_T_, *F*(2,178) = 23.6; η_p_^2^ = 0.21, and V_E_, *F*(2,178) = 35.8; η_p_^2^ = 0.29, all *p*’s < 0.002 (See Table 1). In addition, the time of inspiration (T_I_) and expiration (T_E_) were analyzed. Hypercapnia induced shorter V_I_ without altering V_E_. For the V_i_, an Exposure × Phase interaction, *F*(2,178) = 6.6, η_p_^2^ = 0.07. Although hypercapnia induced quicker inhalation with greater depth, there were no main effects or interactions with BI.

**TABLE 1:** Respiratory and cardiovascular parameters over experiment epochs within ambient air (AA) and 7% CO_2_ conditions in BI and NI participants.

	Baseline	Exposure	Recovery
**AA-NI**			
HR	77.8 ± 2.1	75.5 ± 2.1	77.9 ± 2.1
Mean RR	786.2 ± 20.6	809.9 ± 21.2	784.0 ± 20.6
Breathing frequency (b/min)	13.8 ± 0.7	16.0 ± 0.7*	16.6 ± 0.7
V_T_ (L)	0.9 ± 0.04	0.8 ± 0.04	0.7 ± 0.03
V_E_ (L/min)	11.3 ± 0.5	11.7 ± 0.5	11.5 ± 0.4
T_I_ (s)	2.6 ± 0.1	2.5 ± 0.1	2.4 ± 0.1
T_E_ (s)	1.1 ± 0.1	1.0 ± 0.1	0.9 ± 0.1
**AA-BI**			
HR	78.4 ± 2.7	75.4 ± 2.7	77.8 ± 2.7
Mean RR	777.6 ± 25.9	808.3 ± 26.7	782.6 ± 25.9
Breathing frequency (b/min)	14.2 ± 0.9	17.3 ± 0.8*	17.2 ± 0.8
V_T_ (L)	0.8 ± 0.06	0.7 ± 0.05	0.6 ± 0.04
V_E_ (L/min)	10.6 ± 0.6	10.9 ± 0.6	10.7 ± 0.5
T_I_ (s)	2.7 ± 0.1	1.8 ± 0.1*	2.1 ± 0.1*
T_E_ (s)	1.3 ± 0.1	0.9 ± 0.1	0.9 ± 0.1
**7% CO_2_-NI**			
HR	81.0 ± 2.0	79.6 ± 2.0	81.9 ± 2.0
Mean RR	750.6 ± 19.5	765.6 ± 20.1	745.6 ± 19.5
Breathing frequency (b/min)	13.9 ± 0.7	16.9 ± 0.6*	17.3 ± 0.6
V_T_ (L)	0.9 ± 0.05	1.0 ± 0.04*	0.8 ± 0.03
V_E_ (L/min)	11.2 ± 0.4	15.4 ± 0.5*	13.3 ± 0.3
T_I_ (s)	2.8 ± 0.2	2.3 ± 0.1	2.4 ± 0.1
T_E_ (s)	1.0 ± 0.2	0.8 ± 0.1	1.0 ± 0.1
**7% CO_2_-BI**			
HR	80.8 ± 2.5	80.5 ± 2.5	81.3 ± 2.5
Mean RR	764.0 ± 24.5	765.1 ± 25.3	756.4 ± 24.5
Breathing frequency (b/min)	14.1 ± 0.8	16.5 ± 0.8*	17.5 ± 0.8
V_T_ (L)	0.9 ± 0.06	1.0 ± 0.05*	0.8 ± 0.04
V_E_ (L/min)	11.1±=0.5	15.9 ± 0.6*	13.5 ± 0.4
T_I_ (s)	2.5 ± 0.2	1.8 ± 0.1*	2.0 ± 0.1*
T_E_ (s)	1.0 ± 0.2	0.9 ± 0.1	0.8 ± 0.1

* indicates significant change from Baseline, *p* < 0.05.

### HR and HRV

Analysis of HR only yielded a significant main effect of Phase, *F*(2,178) = 11.5, η_p_^2^ = 0.115. HR generally decreased from Baseline to the Exposure phase. Accordingly, average RR intervals generally increased from the Baseline to the Exposure phase (See Table 1). The exposure phase was coincident with the start of the computerized task, thus general phases changes in HR and RR intervals may reflect attention to the task.

**FIGURE 1:**
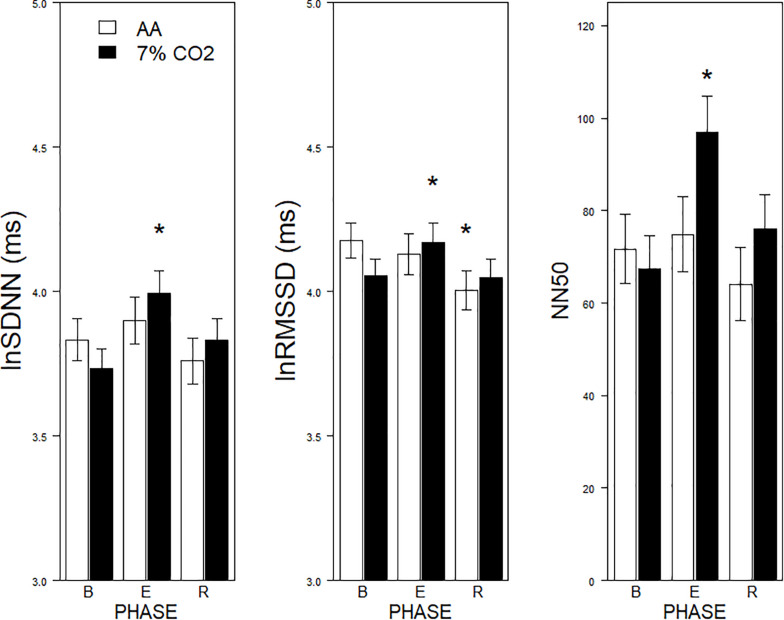
Effects of 7% CO_2_ on measures of Time Domain HRV across the three experimental phases. Three measures of the Time Domain in HRV are depicted from **left to right panels**, SDNN, RMSSSD, and NN50, respectively. The legend for all three panels is contained in the **left panel**. For all three measures, ^∗^ indicates a significant change from baseline, *p* < 0.05. For NN50, ^∗^ additionally indicates a difference in recovery, *p* < 0.05.

For the time domain, Exposure × Phase interactions were evident for SDNN, *F*(2,178) = 4.1, *p* = 0.02, η_p_^2^ = 0.04, RMSSD, *F*(2,178) = 6.5, *p* = 0.002, η_p_^2^ = 0.07, and NN50, *F*(2,178) = 9.7, *p* < 0.001, η_p_^2^ = 0.10 (see Figure 1). For each, HRV increased during hypercapnia whereas increases were not evident in those given AA. For SSDN, the AA group reduced vagal tone across the experimental phases. There were no main effects or interactions with BI.

**FIGURE 2:**
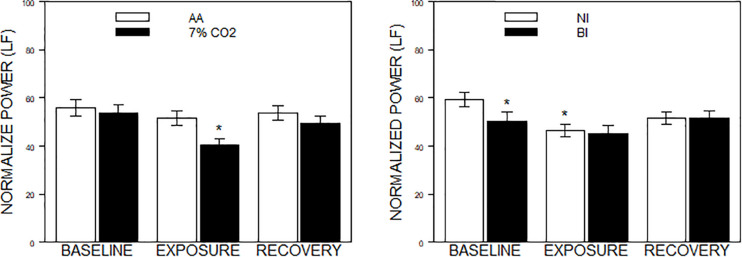
Normalized power in the frequency domain as a function of 7% CO_2_ exposure and temperament. The panels depict measures of normalized power from the HF and LF domains across the three experimental phases. Normalized LF was used for the analysis, but note that LF and HF are reciprocal. The legend for each panel is contained within the panel. The **left panel** depicts the Exposure × Phase interaction in which normalized LF power decreased during 7% CO_2_ exposure which recovered with reintroduction of AA. ^∗^ indicate *p* < 0.05 compared to both baseline values and AA during Exposure. The **right panel** depicts the BI × Epoch interaction. ^∗^ indicate differences relative to the baseline phase, *p* < 0.05. Normalized LF was initially lower in BI than NI. Over participation in the experiment (e.g., computerized task), LF reduced in NI to the levels of BI.

**FIGURE 3:**
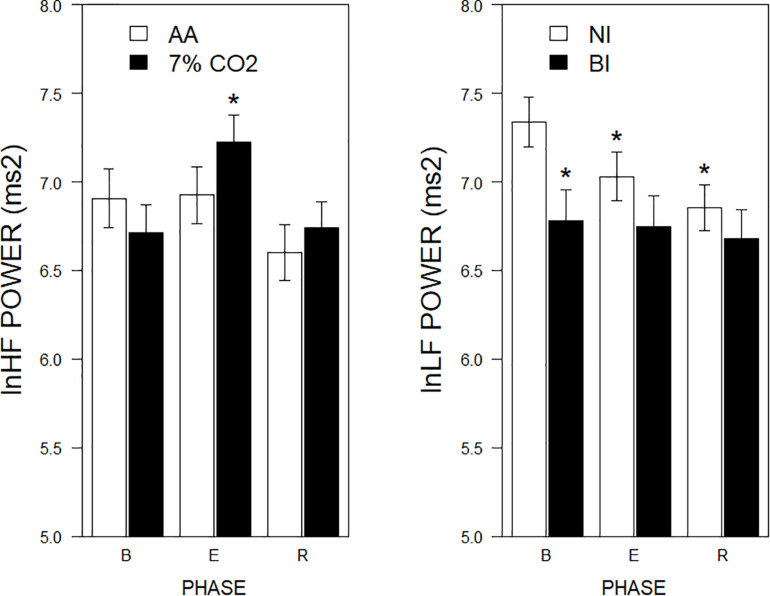
lnLF and lnHF power across experimental phases as a function of 7% CO_2_ exposure and temperament. The **right panel** depicts lnLF power, whereas the **left panel** depicts lnHF power. The legend for each panel is contained within the panel. In the left panel, lnHF power increased in 7% CO_2_ relative to baseline and recovery epochs. lnHF power did not appreciably change in AA over experimental epochs. This pattern is reminiscent of **left panel** of Figure 2. In the **right panel**, lnLF power of BI group was initially lower during baseline than NI. LF power of NI reduced over experimental epochs to be similar to that of BI, which did not change from baseline. This pattern is reminiscent of **right panel** of Figure 2. ^∗^ indicate a significant change from the respective baseline phase, *p* < 0.05.

For the frequency domain, normalized frequencies (nuLF or nuHF) yielded significant interactions of Exposure × Phase, *F*(2,178) = 3.5, *p* = 0.03; η_p_^2^ = 0.04 (See Figure 2, left panel) and BI × Phase, *F*(2,178) = 3.8, *p* = 0.02; η_p_^2^ = 0.04, (See Figure 2, right panel). Interpretation of normalized power requires analysis of the individual lnLF and lnHF power. An analysis of lnHF power indicated an Exposure × Phase interaction, *F*(2,178) = 6.4, *p* = 0.002; η_p_^2^ = 0.07 (See Figure 3, left panel), without significant effects related to BI. This supports the interpretation that differences in normalized power attributable to CO_2_ exposure reflected an increase in HF power. Whereas an analysis of lnLF power showed a BI × Phase interaction, *F*(2,178) = 3.4, *p* = 0.03; η_p_^2^ = 0.034, without effects of Exposure (See Figure 3, right panel). This supports the interpretation that BI × Phase interaction in normalized power was due to decreased lnLF.

### Blood Pressure

Blood pressure, measured before donning the mask and after its removal, did not change as a function of Exposure, BI or time, all *p*’s > 0.35. Mean arterial pressure before the experiment was 92.4 ± 0.9 mmHg and 91.9 ± 1.4 mmHg after.

### Behavior

**FIGURE 4:**
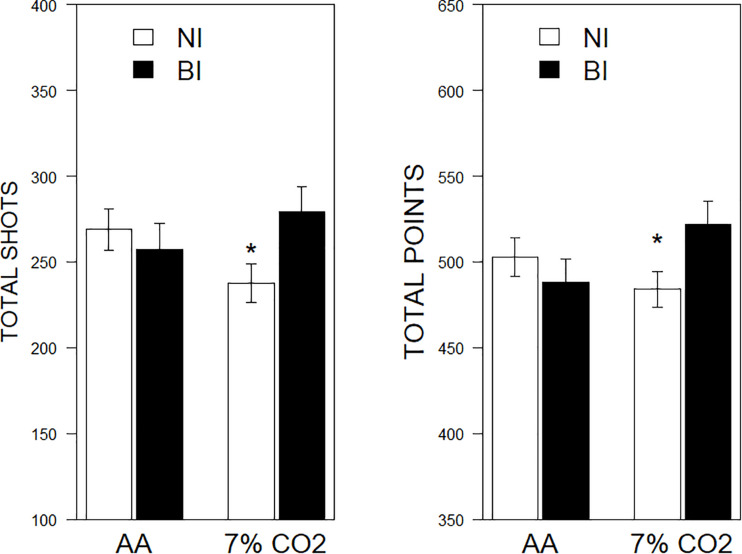
Performance on a computerized game as a function of 7% CO_2_ or AA. The legend is contained within the panels. In the **left panel**, total shots fired are depicted. NI exposed to 7% CO_2_ fired fewer shots than NI given AA and BI exposed to 7% CO_2_. The **right panel** depicts total points scored. Similarly, NI exposed to 7% CO_2_ scored fewer points than NI given AA and BI exposed to 7% CO_2_. ^∗^ indicate a significant change from the respective AA group, *p* < 0.05.

There were significant BI × Exposure interactions for shots fired, *F*(1,81) = 4.04, η_p_^2^ = 0.04, *p* = 0.048, and total points scored, *F*(1,81) = 4.63, η_p_^2^ = 0.54, *p* = 0.034. No significant effects were noted for screen movement. For NI individuals, exposure to CO_2_ tended to inhibit performance, whereas BI individuals exposed to CO_2_ performed better (See Figure 4).

## Discussion

The current protocol examined respiratory and cardiovascular adaptations to moderate hypercapnia in female BI and NI undergraduates while performing an engaging computerized task.

### Task-Related Adjustments

The task, a space-based game, was coincidental with the exposure period. Therefore, changes in respiratory and cardiovascular parameters apparent in both AA and 7% CO_2_ groups are interpreted as adaptations to attending to the task and task performance. Such general changes were apparent as a modest bradycardia and increased respiratory rate. It is against this background that effects from hypercapnia are evaluated.

### Hypercapnia Induces Modest Respiratory Adjustments

Overall, hypercapnia induced modest but robust adjustments in respiratory parameters measured. Hypercapnia increased V_t_ and increased V_E_, compared to the participant’s baseline and relative to those given AA. Ti also decreased during 7% CO_2_ exposure. The pattern of adjustments in respiration parameters are similar to the work of others (Brown et al., 2007; Tzeng et al., 2007), although the magnitude of changes in V_T_ appear less which may reflect differences in the composition of the participant pool.

### Hypercapnia and HRV

Recording of ECG concomitant with respiratory parameters afforded the opportunity to assess the impact of hypercapnia on HRV. The recording parameters were non-optimal (100 Hz sampling frequency over successive 4-min epochs), thus these results need to be understood under this light. Consistent with others, hypercapnia increased nuHF relative to their baseline and the AA group (Brown et al., 2014). Inasmuch as increased nuHF may be a function of increased HF or lowered LF, the individual frequencies were assessed (Laborde et al., 2017). A corresponding interaction showing increased lnHF was apparent, supporting the interpretation that hypercapnia increased HF power. Increased HF power in response to hypercapnia is consistent with the work of others (Brown et al., 2007, 2014). Further, hypercapnia induced increases in HRV in the time domain. An increase in vagal power was apparent with lnSDNN, lnRMSSD, and NN50, with a recovery evident in NN50. The increases were apparent with and without respiration rates included as a covariate in the analyses. The increase in HRV was apparent in the presence of an engaging computerized task that was contemporaneous, albeit not exclusive to hypercapnia. Previous work assessing time domain HRV in moderate hypercapnia have not found differences (Brown et al., 2007, 2014). One potential difference may be the body position of individuals during exposure. In previous studies individuals were supine during exposure; in this study, individuals were seated. Inasmuch as time domain measures of HRV are considerably less in the seated compared to supine position (Young and Leicht, 2011), seated exposure may provide a greater dynamic range to observe increases from hypercapnia.

Hypercapnia did not affect HR. A dissociation between HR and HF HRV in hypercapnia has previously been observed (Brown et al., 2007, 2014) with modest exposure to hypercapnia. Analysis of HRs segregated into inspiratory and expiratory beats found very subtle differences between AA and 5% CO_2_ inhalation (Brown et al., 2007). Although we did not assess RSA, our exposure represents a similarly mild challenge. Further, there are dissociations between HR, HF HRV and respiratory sinus arrhythmia (RSA) during hypercapnia at low levels of exposure (Tzeng et al., 2007; Brown et al., 2014), suggesting a dissociation between vagal tone and HF HRV.

One potential mechanism for increased HF power is acidosis secondary to hypercapnia (Brown and Howden, 2008b). Peripheral chemoreceptors (the carotid bodies and secondarily the aortic bodies) along with central chemoreceptors are sensitive to changes in the blood pH/CO_2_ tension. Evidence suggests that pontomedullary nuclei (medial and lateral parabrachial nucleus, retrotrapezoid nucleus/parafacial area, the medullary raphe, and the pre-Bötzinger complex) are sensitive to pH/CO_2_ tension and signal the phrenic nerve to increase minute ventilation in order to expel excess CO_2_ (Guyenet et al., 2013). The pH/CO_2_ central chemoreceptors drive the increase in activity of the pre-Bötzinger complex (the proposed rhythm generator) eventually stimulating the phrenic nerve, which then drives diaphragm activity, and this increased diaphragm drive ultimately increases breathing. Increased HF power may be secondary to phrenic drive.

Thus, the current protocol and procedures of hypercapnia of 7% CO_2_ delivered over a 4-min period represented a mild physiological challenge.

### BI Temperament

A primary goal of the study was to determine whether BI temperament differed in HRV either as a constituent process and/or as a response to challenge compared to NI individuals. As a constituent process, time domain measures of HRV did not differ between BI and NI individuals. A difference was evident in terms of lnLF power, with BI individuals displaying lower LF power. The difference between BI and NI diminished over experimental phases. Hypercapnia drove LF power lower in NI, however, hypercapnia did not diminish LF in BI suggesting LF power may have been at nadir in BI individuals. An alternative explanation for the lower baseline LF power is BI individuals are more responsive to the laboratory setting, apparatus or instructions therein (a “white coat” reaction). Arguing against this interpretation, HR and BP, two responses normally sensitive to laboratory reactivity, were essentially poolable between NI and BI individuals.

The hypercapnia challenge did not differentially affect the physiological responses of BI participants. The respiratory effects of hypercapnia were no different in BI than NI individuals. Further, there were no exposure-specific differences in HRV in the time or frequency domains. Thus, the inherently low LF power did not manifest in further changes in response to hypercapnia. Note that there was no evidence of panic or panic-like behavioral or physiological responses to hypercapnia in any participant.

A computerized game was presented as a foreground task contemporaneous with CO_2_ exposure. Participants knew that exposure to CO_2_ was possible, but did not know when or if they would be exposed. BI individuals could have perceived hypercapnia as stressful, as more of a challenge than NI individuals. If so, one would expect performance on the foreground task to suffer more than NI performance inasmuch as attention would be divided between the hypercapnia and attendant adjustments and the game. Indeed, hypercapnia mildly degraded NI performance. However, performance of BI individuals exposed to hypercapnia was *better* than hypercapnia-exposed NI participants. Performance was not significantly related to any of the HRV parameters examined.

The enhanced performance of BI individuals is reminiscent of learning performances of BI and animal models of BI. In eyeblink conditioning, the unconditional reflex of BI is of similar magnitude to NI, but learning is facilitated (Holloway et al., 2012; Handy et al., 2018). In avoidance motivated by shock, response to the shock *per se* is similar between models of BI and NI, but avoidance is facilitated (Servatius et al., 2008). While primary physiological responses to challenges may be similar between BI and NI, the motivational properties to alter behavior largely distinguish BI and NI and by extension vulnerability to develop anxiety disorders. In the face of challenges, BI individuals marshal behavioral and by extension experiential resources. Displacement and focused attention, as exhibited by BI individuals, may be fundamental to anxiety vulnerability. Inherently low LF power may provide the physiological basis for biased performance under modest challenge.

### Limitations

There are several limitations to consider in evaluating the results from the current study. As stated earlier, the parameters for assessing HRV were not optimal. It is recommended that epochs for LF power assessments are 5 min and sampling rate for ECG is 200 Hz (Quintana and Heathers, 2014; Laborde et al., 2017). The finding of inherently low LF power in BI should be replicated with more optimal parameters. The HF component and the time domain measures are well within recommendations. Accordingly, the observed increased HF during hypercapnia is consistent with that in the literature regarding the impact of moderate hypercapnia on HRV (Brown et al., 2007, 2014). It should also be noted that we restricted the study to female participants. Females are generally an understudied population in psychophysiology. In the literature cited concerning hypercapnia and HRV, females comprise about 25% of those samples with numbers too small to know whether males and females differ in reactivity to hypercapnia. A study with male participants to directly compare and extend the findings is warranted. We also did not record respiration as an independent channel precluding an analysis of RSA. Similarly, blood gases were also not monitored. Both of these measures were challenging within the small undergraduate college environment with the resources available.

### Summary

In summary, mild hypercapnia induces increases in HF power consistent with the work of others. However, we also observed increased HRV in the time domain in response to 7% CO_2_ particularly with the measures of RMSSD and NN50. A primary purpose was to evaluate HRV in BI as well as in response to the 7% CO_2_ challenge. A trait-like reduction in LF power was observed in BI females. Moreover, BI females displayed enhanced reactivity to 7% CO_2_ with improved performance on the computerized task. However, the respiratory responses to hypercapnia as well as HRV reactivity were similar between BI and NI. To the degree which inherently lower LF power contributes to vulnerability needs to be elaborated. These data contribute to an understanding of vulnerability to develop anxiety disorders in keeping with a learning diathesis model.

## Data Availability Statement

The raw data supporting the conclusions of this article will be made available by the authors, without undue reservation.

## Ethics Statement

The studies involving human participants were reviewed and approved by the Carthage College Institutional Review Board. The patients/participants provided their written informed consent to participate in this study.

## Author Contributions

RS, PM, and DM performed the design of the experiment. PM, DM, JM, and DC-S were responsible for IRB interactions and data collection. PM, DM, and JM accomplished the instrumentation. PM, DM, JM, and DC-S accomplished the data collation. RS, JH, and MA accomplished the data analyses. All authors contributed to manuscript preparation and editing.

## Disclaimer

The views expressed in this article reflect the results of research conducted by the authors and do not necessarily reflect the official policy or position of the Department of Veterans Affairs, Department of the Navy, Department of Defense, nor the United States Government. RS and JH are employees of the United States Government. This work was prepared as part of their official duties. Title 17 U.S.C. §105 provides that “Copyright protection under this title is not available for any work of the United States Government.” Title 17 U.S.C. §101 defines a United States Government work as a work prepared by a military service member or employee of the United States Government as part of that person’s official duties.

## Conflict of Interest

The authors declare that the research was conducted in the absence of any commercial or financial relationships that could be construed as a potential conflict of interest.
